# Shear Performance Assessment of Sand-Coated GFRP Perforated Connectors Embedded in Concrete

**DOI:** 10.3390/ma12121906

**Published:** 2019-06-13

**Authors:** Zhihua Xiong, Yuqing Liu, Yize Zuo, Haohui Xin

**Affiliations:** 1 Department of Bridge Engineering, Tongji University, 1239 Siping Road, Shanghai 200092, China; xiongzhihua_2013@126.com (Z.X.); yql@tongji.edu.cn (Y.L.); 2 China Construction First Division Group Construction and Development Co., Ltd., Beijing 100161, China; zuoyize97@126.com; 3 Faculty of Civil Engineering and Geosciences, Delft University of Technology, 2600 AA Delft, The Netherlands

**Keywords:** GFRP, sand coated, shear behavior, concrete, pull-out test

## Abstract

In order to evaluate the shear performance of sand-coated glass fiber-reinforced polymer (GFRP) perforated connectors (SCGPC) embedded in concrete, 8 pull-out tests were conducted. Finite element (FE) analysis considering GFRP failure and cohesion between GFRP and concrete of SCGPC were conducted for parametric analysis. Effects of surface treatment, hole’s radius, embedment length, and multi holes were examined. The test and theoretical analysis revealed that the strength of SCGPC is considerably larger than GFRP Perforated Connector (GPC). The stiffness of SCGPC is determined by the adhesion between concrete and GFRP. When GFRP plate’s thickness is less than the critical thickness, the embedment length plays a major role in the strength of SCGPC. When embedment length is less than the effective bond length, the shear strength of SCGPC is governed by both the adhesion and GPC’s shear capacity; otherwise, the strength of SCGPC is governed by the adhesion strength. Furthermore, an empirical equation was suggested to predict the shear strength of SCGPC. The equation involves the failure mechanism of both bond and GPC and deals the strength of SCGPC into two ranges according to the embedment length. Good agreement was achieved between the strength prediction by the suggested equation and the parametric analysis result.

## 1. Introduction

Glass fiber-reinforced polymer (GFRP)-concrete composite structures have been widely used in infrastructure [1,2,3,4,5]. The connection between GFRP and concrete is of great importance. The types of connection for GFRP-concrete composite structures includes bolt, sand-coated, and perforated. Since casting concrete directly on GFRP formwork results in poor bonding performance [6], a sand-coated GFRP profile is a good option to increase the combination between concrete and GFRP. Cho et al. tested a sand-coated GFRP-concrete bridge deck with bolted connection [7], the fatigue performance of sand-coated GFRP-concrete deck was significantly improved by the presence of the bolt. Woltman et al. [8] investigated the pull-out performance of a shear connector made of sand-coated GFRP rebars which presented a higher strength than the specialized polymer connector. However, it has been reported that the humid environment may cause bond degradation [9]. In the meantime, a perforated plate connection is widely used and preferred in steel-concrete composite structures [10,11]. The combination of sand-coated surface and perforated plate has been tested in composite slabs by the authors [12]. It has been found that the flexural resistance of slabs with sand-coated GFRP perforated connectors (SCGPC) was much larger than that of slab without surface treatment. It is still unclear about the improvement of the shear capacity of SCGPC than that of plate without surface treatment.

Generally, the bond behaviors between sand-coated fiber-reinforced polymer (FRP) plate and concrete are characterized by bond strength, effective bond length, and load-slip curve. In FRP strengthening concrete structures, concrete strength has been found to have a substantial effect on the adhesion strength. Chen and Teng developed a model based on nonlinear fracture mechanics and test data for the externally bonded FRP [13] in which the ultimate adhesion strength is proportional to the square root of concrete cylinder compressive strength *f*_c_’. Seracino [14] proposed a generic model considering both externally bonded FRP sheets/plates and near-surface mounted FRP strips, in which the ultimate adhesion strength is proportional to *f*_c_’^0.33^ similar as Bilotta [15]. Kalupahana [16] carried out a series of 44 near-surface mounted carbon fiber-reinforced polymer (CFRP) tests indicating that the concrete strength effect on the ultimate strength depends on the FRP’s surface configuration and the embedment length. 

Effective bond length *L*_e_ (also called critical bond length) is a definition of FRP’s embedment length at which the adhesion resistance will reach the maximum and will not develop as the length enlarges [13]. Seracino suggested a minimum 200 mm effective bond length for near-surface mounted CFRP by pull-out tests [17]. Naser et al. conducted a series of tests and numerical investigations on the optimum embedment length of CFRP laminate [18]. As regard to the load-slip curve, it is a reflection of interfacial traction-separation constitution. The push/pull-out tests of sand-coated FRP plate/rebar, finite element (FE) simulation is a regular tool to investigate the bond behavior [19]. Cohesive zone modeling (CZM) is one of the most common methods in FE simulation, such as: Chen and EL [20] used CZM to obtain mixed-mode bending fracture energy of the interface between sand-coated GFRP and UHPC; and Tekle et al. [21] implemented CZM to investigate strand and bond distribution along the sand-coated GFRP bars. Besides, the tests about the roughness of sand-coated surface have been done to understand its effect on adhesion strength, however, the result showed a scatter about the adhesion resistance and the aggregate size [22,23]. 

In terms of GFRP perforated connectors (GPC) embedded in concrete, push/pull-out tests have been reported. Based on the experimental findings, Cho et al. [24] proposed a strength equation on the basis of discrete spring model; Zou et al. [25] proposed a strength equation on the basis of the Oguejiofor and Hosain Model; and the authors [26] proposed a strength equation considering the effect of stress concentration of GFRP hole. 

Although plenty of previous tests and analysis have been performed on sand-coated FRP joint and GPC, SCGPC involves the interaction between the perforated GFRP plate and concrete, knowledge on the shear behavior of SCGPC is limited as far as the authors’ knowledge. Furthermore, the shear capacity of SCGPC or the comparison between SCGPC and GPC, these issues are quite important yet haven’t been reported. This paper carried out experimental and numerical investigations to reveal the failure mechanism of SCGPC and to understand the shear behavior of SCGPC. The novelty of this research embodied in the following aspects: (1) the shear failure mode of SCGPC was found and compared to that of GPC, which provided a reference to the structural design; (2) FE model of SCGPC based on lamina theory was built and validated by pull-out test. CZM was incorporated in the FE model and presented a precise capture of the bond behavior of SCGPC; and (3) an empirical equation was proposed to predict the strength of SCGPC.

## 2. Pull-Out Tests 

### 2.1. Materials

**Figure 1:**
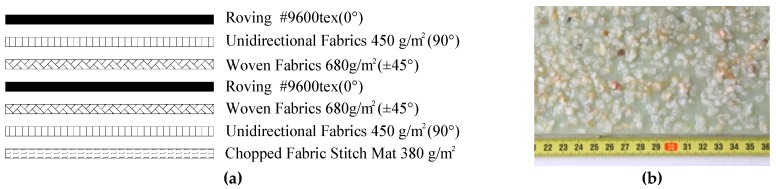
Sand-coated glass fiber-reinforced polymer (GFRP) plate. (**a**): Layers of pultruded GFRP plate; (**b**): Sand-coated treatment surface, (unit: cm).

The pultruded GFRP plates were used in the pull-out tests. The pultruded GFRP lamination was made up of 7 layers, in which reinforcements were E-glass roving, woven fabrics, and a matrix of epoxy resin. The stacking sequence is shown in Figure 1a, with four types of laminas: (1) rovings for 0° lamina; (2) unidirectional fabrics for the 90° lamina; (3) woven fabrics for the ±45° lamina; and (4) chopped fabric mats. The angles were relative to the pultrusion direction. The material characteristics of pultruded GFRP laminations were obtained by the coupon tests and summarized in Table 1, where the tensile, compressive, and shear strength of the GFRP plate was tested according to Chinese code GB1447-2005, GB1448-2005, and GB/T28889, respectively. Epoxy adhesives and the coarse silica sand aggregates with a range of 4–7 mm were used in the fabrication of sand-coated interface.

Three 150 mm edge concrete blocks for compression test were poured and cured in moisture environment for 28 days. The material properties of concrete were tested in accordance with Chinese code GB50010-2010 and a cube strength of 50.9 MPa was reported by the authors [26]. In addition, the elastic modulus and yield strength of the steel rebars were 2.05 × 10^5^ MPa and 430.4 MPa, respectively [26].

### 2.2. Fabrication of Pull-Out Specimens

**Figure 2:**
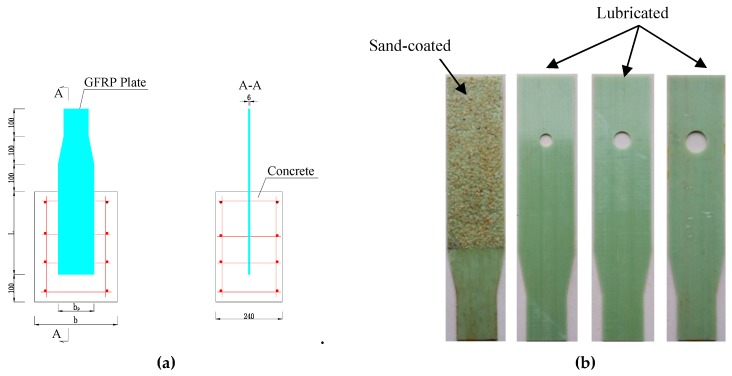
Specimen’s layout. (**a**): Layout of specimen; (**b**): Pultruded GFRP plates.

**Figure 3:**
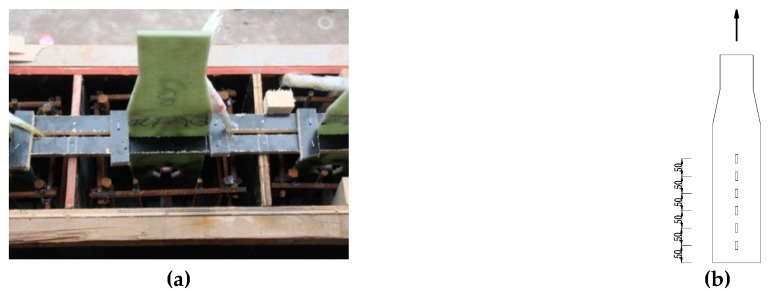
Specimen’s fabrication. (**a**): Fabrication process; (**b**) Strain gauges on SC-P, (unit: mm).

The pull-out specimens were divided into two groups. The first group included two sand-coated GFRP plates; the second group included three types of GPC [26]. As shown in Figure 1b, the bonding interface between the GFRP plate and concrete was first filtered by manual sandpaper. A thin layer of epoxy was then applied to the surface of GFRP plate. The coarse silica sand aggregates were evenly pressed onto the plate’s surface. Prior to casting, the plates with sand-coated surface were cured undisturbed for seven days. As presented in Figure 2, the general layout of the first group and the second group were the same except the second group contained a hole and its surface treatment. The gripped end of specimen was tapered to 90 mm and the width embedded in concrete was still 130 mm to meet the width demand of the grip. GFRP plates were pre-installed in the wood formwork as shown in Figure 3. The plate’s surface of the second group was lubricated to reduce the friction with concrete. After the deployment of the strain gauges as shown in Figure 3b, the concrete was poured to the formwork presented. Each type of test contained two specimens. The geometric parameters of the pull-out specimens are listed in Table 2, where *R* is the hole’s radius; *b* is concrete block’s width; *b*_p_ is GFRP plate’s width and *L* is embedment length. 

### 2.3. Pull-Out Test Setup

**Figure 4:**
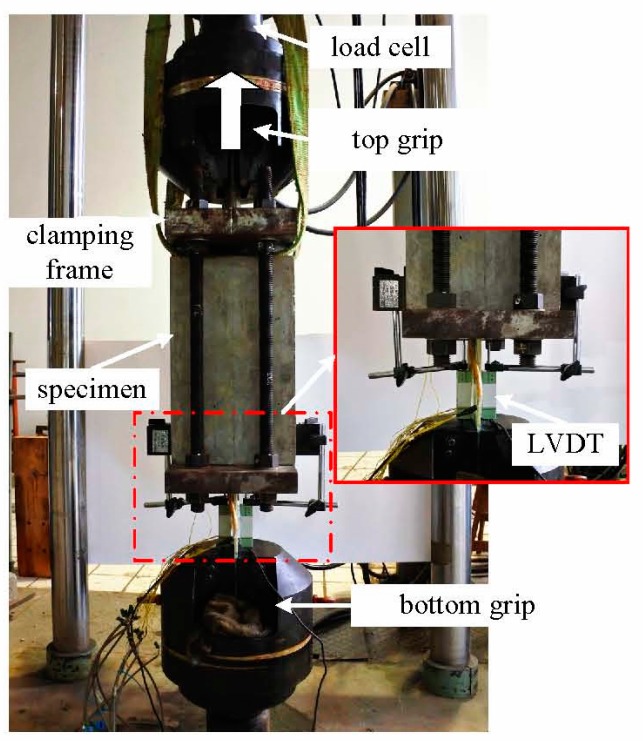
Test set-up (MTS, Shanghai, China).

In Figure 4, there was a clamping frame consisted of rigid plates, bolt shanks, and pinned bar. The specimens were fixed in the clamping frame and the narrowed end of GFRP plates was gripped by the bottom clip. The pinned bar of the clamping frame was gripped by the top clip and pulled by a ±500 kN actuator, with a loading rate 1 mm/min. Before the test, a preloading was applied in a ratio of 10% peak load to check the operation of the gauges on the sand-coated specimens. 

### 2.4. Pull-Out Test Results

#### 2.4.1. Sand-Coated Specimens Result

**Figure 5:**
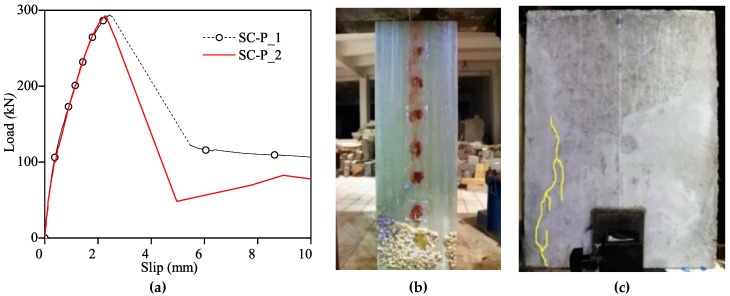
Specimen SC-P test’s result. (**a**): load-slip curves. (**b**): GFRP plate’s failure pattern. (**c**): loaded end crack.

Specimen SC-P was observed to reach its peak load in a short while and dropped rapidly in post-peak branch as shown in Figure 5a. Along the loading process, a rush sound was heard when the load reached to the peak. Finally, the whole sand-coated plate was pulled out. In Figure 5b, it presented interfacial adhesion failure with some micro-crack on the loaded end of concrete block as shown in Figure 5c. A part of concrete near the loaded end was pulled and the crack was caused as presented in Figure 5c, leading to uneven bond strain distribution. 

**Figure 6:**
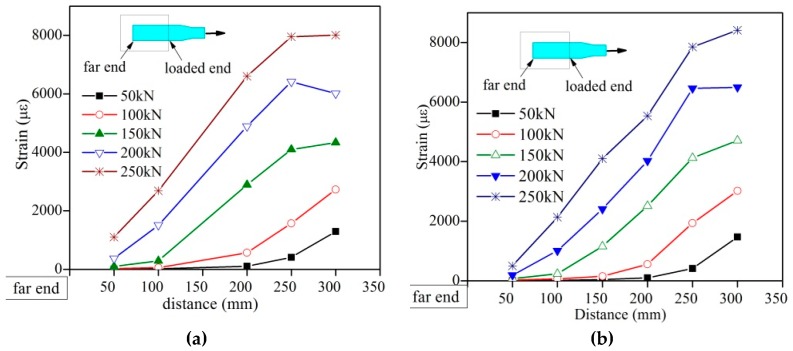
SC-P’s strain distribution. (**a**): SC-P_1; (**b**) SC-P_2.

As shown in Figure 6, in the initial loading stage, the loaded end strain developed rapidly while the far end didn’t bear the force, where the end of concrete block next to the gripped side denoted as the “loaded end” and the end of concrete block far to the gripped side denoted as the “far end”. As the load increased, the force bearing area gradually grew to the far end. Generally speaking, the loaded end held the most part of the pulling force till the specimen’s failure. 

#### 2.4.2. GPC Result

**Figure 7:**
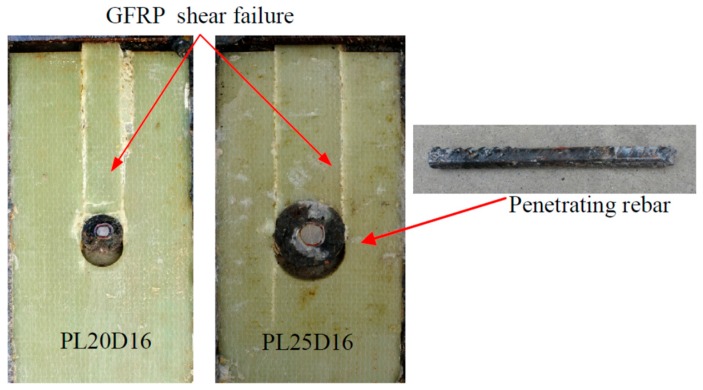
GPC’s failure pattern.

**Figure 8:**
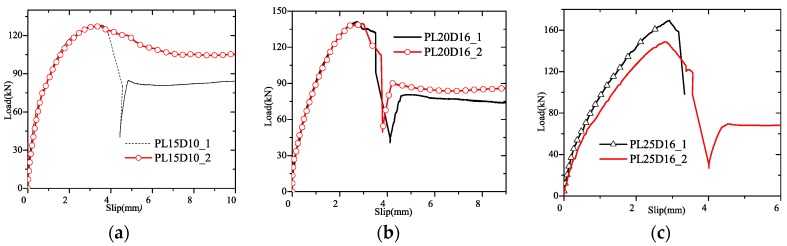
Load-slip curves of GPC. (**a**): PL15D10; (**b**): PL20D16; (**c**) PL25D16.

In Figure 7, by cutting the specimens along the interface between GFRP and concrete, the shear failure of GFRP plate was observed. The penetrating rebar did not yield. The shear resistance ascended as the hole’s radius increased as shown in Figure 8. In terms of slip, it is found that the ductility of GPC is larger than SC-P.

As indicated in the above context, the sand-coated plate is very brittle during pulling, while GPC’s shear strength is lower than sand-coated specimens. For GPC, the shear strength increased when *R* became from 15 mm to 25 mm.

## 3. Sand-Coated GPC Numerical Analysis 

In Section 2, sand-coated plates and GPC were tested separately. Here, SCGPC has been investigated through numerical method. Firstly, pull-out tests have been simulated by finite element model created by ABAQUS. With the validation of FE models, SCGPC FE model has been built and analyzed to reveal its failure mechanism.

### 3.1. Description of the Model and Its Verification

**Figure 9:**
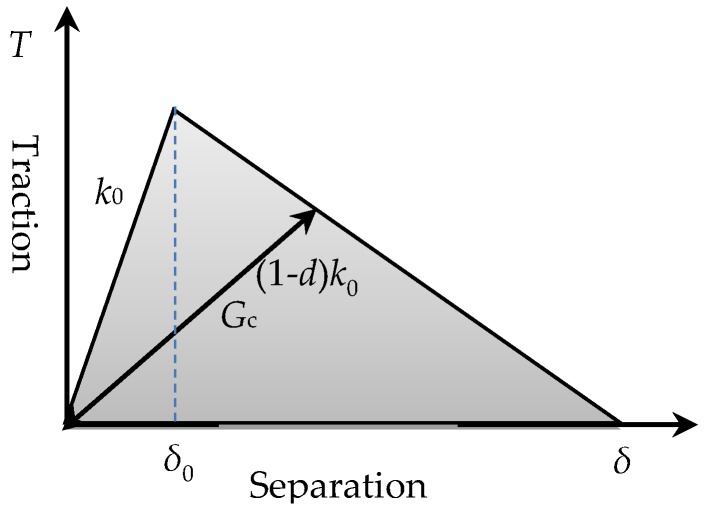
Cohesive behavior.

Cohesive behavior is widely used in the modeling of the bond-slip relationship between FRP and concrete [19]. Considering the fairly small thickness of the bond interface, the surface-based cohesive method was adopted in this article. This behavior assumes a linear elastic traction-separation law before damage and displays progressive degradation of the cohesive stiffness once exceeding the initial damage criteria, as shown in Figure 9. The uncoupled separation and stress relation is expressed as Equation (1). The stiffness coefficient was determined as: *k*_nn_ = 100*k*_ss_ = 100*k*_tt_ according to [27]. Maximum bond stress was derived from the pull-out test with the assumption that 80% shear area reached its maximum bond stress at peak load. The damage initiates when the interfacial stress reaches its maximum bond stress and is expressed as: Max{*t*_s_/*t*_s_^0^, *t*_t_/*t*_t_^0^} = 1. Fracture energy *G*_c_ of cohesive behavior was adopted as 6.91 N· mm^−1^ according to [14]. To overcome convergency problem, a viscosity coefficient of 0.0001 was assumed.

(1) $$T=\left(\begin{array}{c} t_{n}\\ t_{s}\\ t_{t} \end{array}\right)=\left[\begin{array}{ccc} \begin{array}{c} k_{nn}\\ 0\\ 0 \end{array} & \begin{array}{c} 0\\ k_{ss}\\ 0 \end{array} & \begin{array}{c} 0\\ 0\\ k_{tt} \end{array} \end{array}\right]\left(\begin{array}{c} \delta_{n}\\ \delta_{s}\\ \delta_{t} \end{array}\right)=K\delta$$

Hashin’s failure theory [28] was considered in the analysis. This criterion consists four different damage initiation mechanisms, namely; (1) fiber tension (ft), (2) fiber compression (fc), (3) matrix tension (mt), and (4) matrix compression (mc) as expressed in Equations (2)–(5).

(2) $$F_{ft}={\left(\frac{\sigma_{11}}{X_{T}}\right)}^{2}+{\left(\frac{\sigma_{12}}{S_{L}}\right)}^{2}=1\;\sigma_{11}\geq 0$$

(3) $$F_{fc}={\left(\frac{\sigma_{11}}{X_{C}}\right)}^{2}=1\;\sigma_{11}<0$$

(4) $$F_{mt}={\left(\frac{\sigma_{22}}{Y_{T}}\right)}^{2}+{\left(\frac{\sigma_{12}}{S_{L}}\right)}^{2}\;\sigma_{22}\geq 0$$

(5) $$F_{mc}={\left(\frac{\sigma_{22}}{2S_{T}}\right)}^{2}+\left[\frac{Y_{C}}{2S_{T}}-1\right]\frac{\sigma_{22}}{Y_{C}}+{\left(\frac{\sigma_{12}}{S_{L}}\right)}^{2}\;\sigma_{22}<0$$

where *σ*_ij_ are the components of the true stress tensor, *X*_T_ is the longitudinal tensile strength, *X*_C_ is the longitudinal compressive strength, *Y*_T_ is the transverse tensile strength, *Y*_C_ is the transverse compressive strength, and *S*_L_ and *S*_T_ are the longitudinal and transverse shear strengths, respectively. The ultimate strength of lamina is summarized in Table 3 based on the authors’ previous work [29]. Damage initiation properties of FRP lamina were calculated based on micromechanics.

Pultruded GFRP profile used in infrastructure usually has a large thickness and its ply thickness is not as precise as that in aerospace application. A practical method to predict ply thickness has been proposed by authors [30], the GFRP plate’s predicted ply thickness is summarized in Table 4. 

The damage plasticity model in ABAQUS was used as the material constitution of concrete. FIB model was adopted as the stress-strain relationship of concrete [31]. For concrete compression, uniaxial stress-strain curve was determined by Equation (6). For concrete tension, two bilinear approaches expressed in Equations (7) and (8) were used.

(6) $$\frac{\sigma_{c}}{f_{cm}}=\frac{k\eta -\eta^{2}}{1+(k-2)\eta }$$

(7) $$\sigma_{ct}=f_{ctm}\left(1-0.8\frac{w_{t}}{w_{tc}}\right)\;w_{t}\leq w_{tc}$$

(8) $$\sigma_{ct}=f_{ctm}\left(0.25-0.05\frac{w_{t}}{w_{tc}}\right)\;w_{tc}<w_{t}<w_{tf}$$

where, *σ*_c_ and *ε*_c_ are the stress and strain at any point on the compressive curve; *k* = 0.464·*E*_c0_ · *ε*_c1_ ·(*f*_cm_)^−2/3^; *f*_cm_ and *ε*_c1_ are the maximum compressive stress and its corresponding strain; *η* = *ε*_c_/*ε*_c1_; *ε*_c1_ = 0.0026; *σ*_ct_ is the stress at any point on the tensile curve; *w*_t_ is crack opening; and *w*_tc_ = *G*_f_ /*f*_ctm_. Dilation angle of concrete to control the plastic flow was assumed as 38° according to Jankowiak [32].

**Figure 10:**
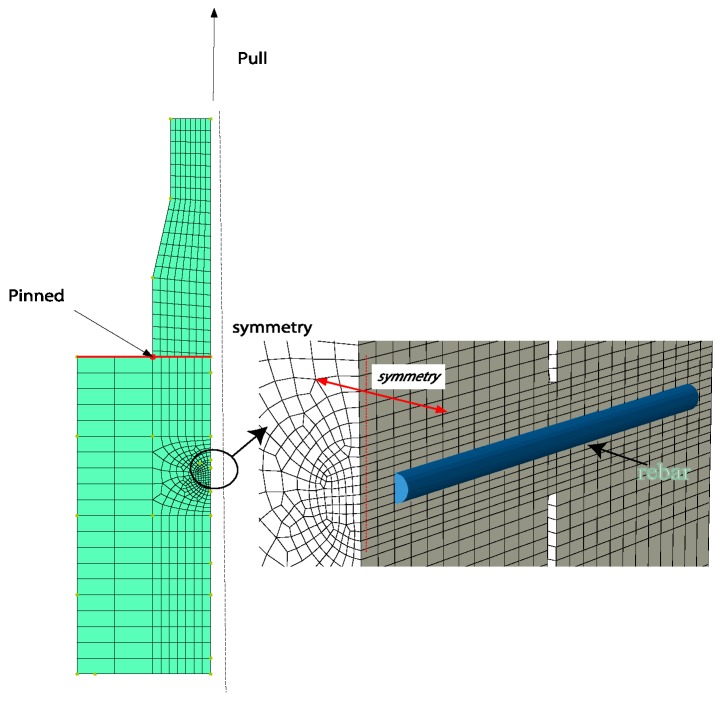
Finite element (FE) model.

FE models were then built to simulate the failure process of specimens SC-P and PL20D16. The model was built in a half due to symmetric condition and pinned constraints were applied on the loaded end of specimen as presented in Figure 10. Solid element (C3D8R) and continuum shell element (SC8R) were selected as the element type for concrete and GFRP plate, respectively.

**Figure 11:**
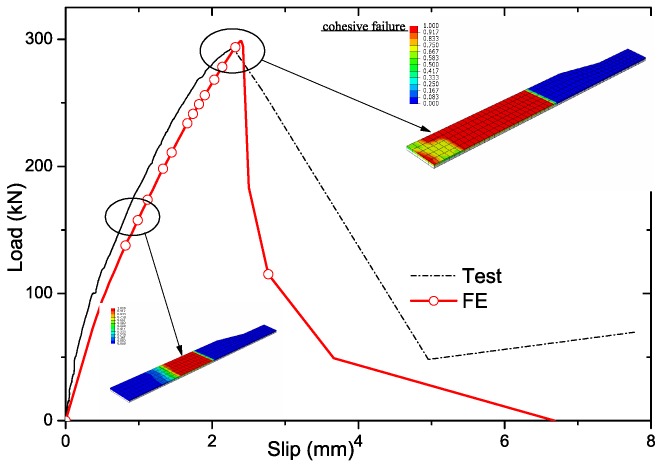
Verification of sand-coated specimens’ FE model.

**Figure 12:**
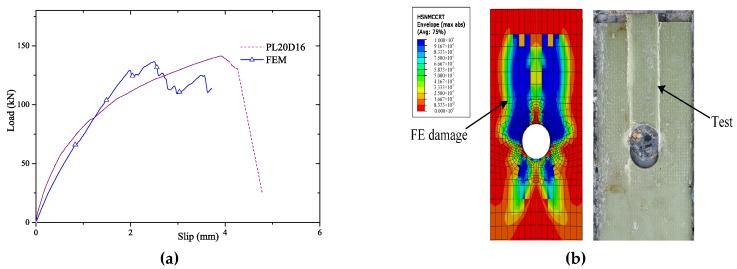
Verification of GFRP perforated connectors (GPC)’ FE model. (**a**): Verification of GPC’ FE model; (**b**): Failure pattern by FE vs. the test.

As presented in Figure 11 and Figure 12a, the load-slip curves calculated by FE of both sand-coated plate and GPC agreed well with the test specimens SC-P and PL20D16. The debonding process of SC-P by FE matched the findings of pull-out test, which indicated the bonding failure initiated in the loaded end of plate and developed gradually to the far end as presented in Figure 6 and Figure 11. In Figure 12b, the ultimate damage state of FE captured the shear failure mode of GFRP plate as well.

### 3.2. Parametric Analysis

Since the FE model has been proven its accuracy in simulating the failure of sand-coated specimens and GPC, a series of SCGPC FE models were built to investigate its shear failure mechanism. The modeling strategy and material constitutions were similar as described in Section 3.1 except that the penetrating rebar was not considered in the parametric analysis. The format of numbering of the specimens in Table 5 is: NS—non-sand-coated; R—radius of hole; SP—sand-coated plate; M—multi hole; E—embedment length. For multi-hole specimens, the plate’s width *b*_p_ = 220mm, the two hole’s center distance is 100 mm.

## 4. Parametric Analysis Result

**Figure 13:**
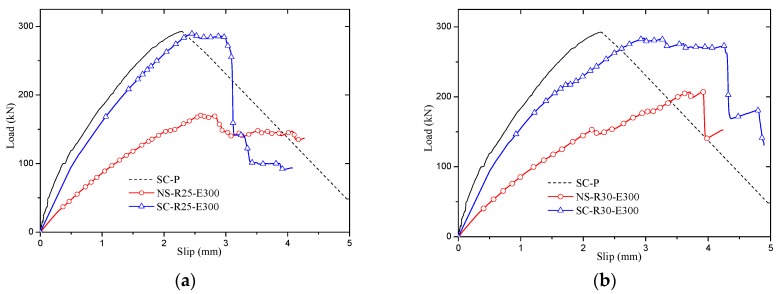

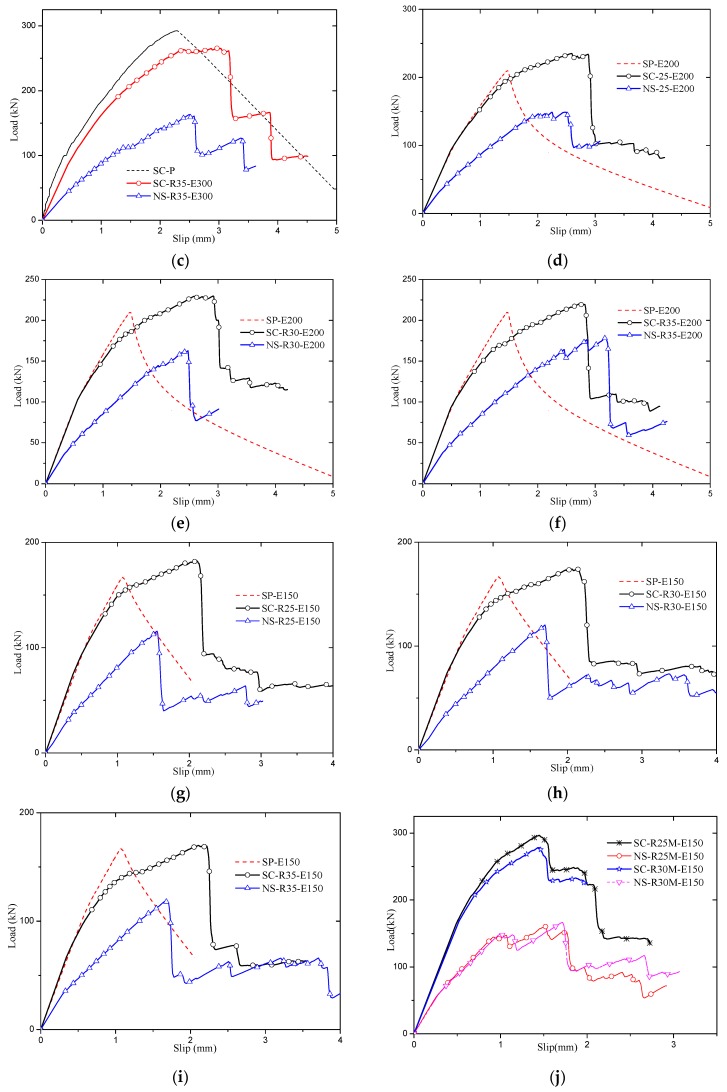
Load-slip curves of sand-coated GFRP perforated connectors (SCGPC). (**a**) R = 25, E = 300; (**b**) R = 30, E = 300; (**c**) R = 35, E = 300; (**d**) R = 25, E = 200; **(e)** R = 30, E = 200; (**f**) R = 35, E = 200; (**g**) R = 25, E = 150; (**h**) R = 30, E = 150; (**i**) R = 35, E = 150; (**j**) Multi hole.

The load-slip curves of parametric analysis are plotted in Figure 13. It is found that the SCGPC’s shear capacity is considerably larger than that of GPC, and the SCGPC is more ductile than the sand-coated plate when bearing load. The load-slip curves of SCGPC presents a similar yield plateau especially when the embedment length is larger than critical value as Figure 13a–c.

**Figure 14:**
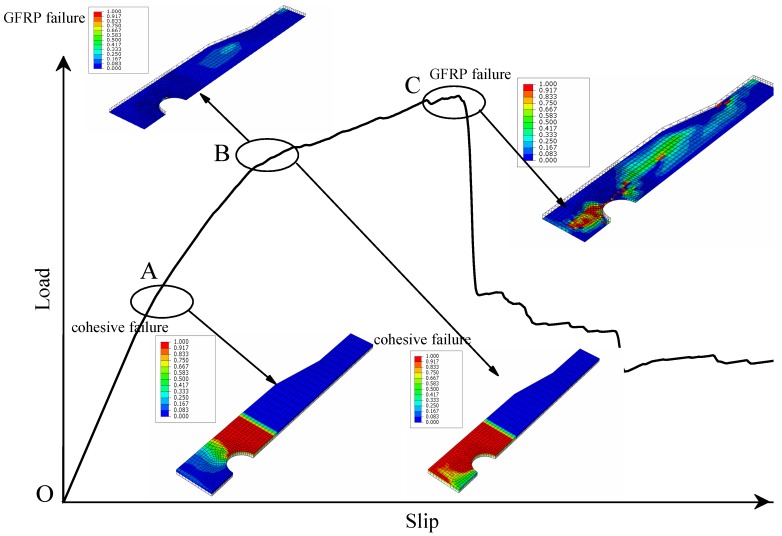
Generalization of SCGPC failure process.

By generalizing the load-slip curves of Figure 13d–i, the failure process can be illustrated as Figure 14. At the initial stage from point O to point A, the adhesion holds the contact force between the plate and concrete and degrades swiftly as the cohesive damage contour reflects. From point B to point C, the bond resistance drops rapidly and GFRP plate starts to damage. When reaching the peak load point C, GFRP plate appears large area of matrix compressive damage. Finally, the plate presents the shear failure as GPC. 

With the knowledge of the failure process as shown in Figure 14, two points are understood about SCGPC specimens of embedment length 150–200 mm, (i) the bond failure is prior to the GFRP shear failure; and (ii) the plate’s failure pattern is the same as that of GPC. According to previous research [26], for the plate shear failure of GPC, there is a critical plate thickness *t*_cr_. When the plate is thinner than *t*_cr_, the plate tends to appear shear failure, on the contrary, GPC turns up concrete dowel failure. According to the prediction in [26], plate thickness of GPC with the same dimension should be at least 10 mm to meet the requirement of *t*_cr_, therefore, 6 mm thickness is thinner than *t*_cr_. In other word, the plate’s failure pattern of SCGPC fits the theory of shear failure of GPC.

Next, the parameters affecting the shear capacity of SCGPC have been addressed separately, which included embedment length, radius, multi-hole, and penetrating rebar.

**Figure 15:**
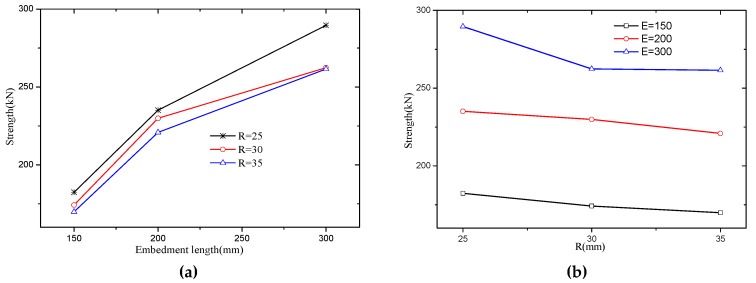
Parameter’s effect on strength of SCGPC. (**a**): Embedment length; (**b**): Radius.

Firstly, the embedment length indicated a significant impact on the shear strength of SCGPC as shown in Figure 15a. Especially when radius is fairly small like *R* = 25 mm, SCGPC behaved as similar as sand-coated plate. In terms of the previous literature [13], shear strength of external bonding of GFRP won’t improve significantly if the embedment reaches its effective bond length. According to Cheng and Teng model [13], effective bond length *L*_e_ can be determined as Equation (9), where *f*_c_’ and *E*_p_ are concrete cylinder strength and FRP’s elastic modulus, respectively. However, the sand-coated embedment of SCGPC is analogous to the near-surface mounted strip, its debonding mechanism is different from the external bonding [14]. Substituting the material property in this research to Equation (9), *L*_e_ = 296 mm. For the type of E-300 specimens, the embedment length *L* is larger than *L*_e_. It can also be observed from Figure 15a, when embedment length ascended from 200 to 300mm, the secant of the curves all became flatter than the initial stage.

On the mean time, concrete strength actually is another major factor affecting the strength of SCGPC. Including the definition of effective bond length *L*_e_, GFRP plate’s critical thickness *t*_cr_, and the empirical equation of SCGPC’s shear strength in the next section, concrete strength has been all involved in. Therefore, it is not discussed alone in this article.

(9) $$L_{e}=\sqrt{\frac{E_{p}t}{\sqrt{f_{c}{}^{\prime }}}}$$

As regard to the effect of hole’s radius, it is presented in Figure 15b. When embedment length is deep, the strength of SCGPC decreased with the increasing radius. This is attributed to the discounted bond capacity due to the perforated area. Hole’s radius makes insignificant effect on SCGPC’s strength when embedment length is short like E = 200 mm. Although the bond capacity decreases as the radius increases, the counteraction of the ascending strength of GPC makes the strength of SCGPC stay still.

With respect to the effect of multi-hole, the double-hole specimen’s strength was less than two times of single hole specimen, this could be owing to larger stress concentration. Comparing the strength of NS-R25-E300 and test specimens SC-R25, it was found the penetrating rebar didn’t play a role in the GFRP shear failure, which has also been reported by the previous test [26]. 

## 5. Failure Mechanism and Empirical Equation

**Figure 16:**
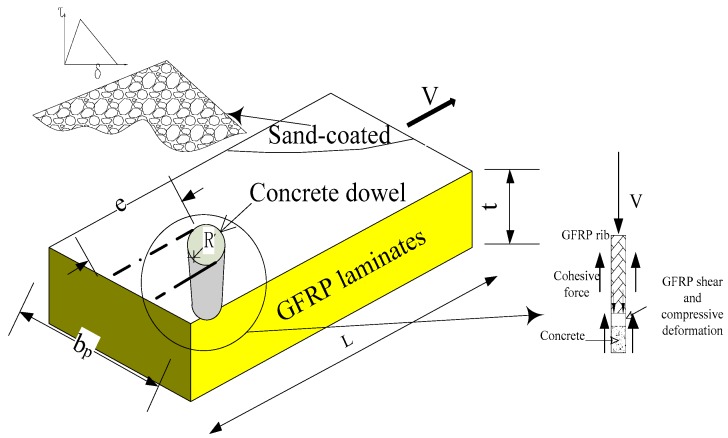
Failure scheme of SCGPC.

Through the parametric analysis and its discussion, when GFRP plate’s thickness is less than the critical thickness *t*_cr_, SCGPC’s failure mode can be classified into two situations: (1) bond failure governing if *L* ≥ *L*_e_; and (2) a mixture of bond failure and GFRP shear failure if *L* < *L*_e_. In the first circumstance *L* ≥ *L*_e_, the bond resistance governs the strength of SCGPC like SC-E300 in Figure 13a–c, while the strength of SCGPC is less than the pure sand-coated plate since there is perforated area. In the second circumstance *L* < *L*_e_, sand-coated surface of SCGPC debonds initially and then the GFRP plate bears the shear load and fails at last as shown in Figure 14. In Figure 13, the stiffness of the SCGPC is obviously larger than that of GPC and is determined by the bond strength. The failure process and mechanism are illustrated in Figure 14 and Figure 16, respectively.

According to the failure mechanism of SCGPC, an empirical equation is suggested. The empirical equation predicts the strength in two regions by the value of *L*. When *L* ≥ *L*_e_, it is assumed that the strength of SCGPC is governed by the bond resistance given by Equation (10), which has been proposed by Seracino [14] and is modified to consider the reduction effect caused by the perforated area. In Equation (10), *L*_per_ = 2(*L*+1)+(*b*_p_+1); *ϕ*_f_ = (*L*+1)/( *b*_p_+1); *A*_p_ is FRP’s shear area, *f*_rup_ is FRP’s rupture stress; all the length unit is millimeters; and when *L* < *L*_e_, it is assumed that the strength is composed of post-peak resistance of the adhesion and the GPC’s shear resistance *V*_GPC_, which is then written as Equation (11). In Equation (11), *V*_GPC_ has been reported as Equation (12) [26], effective bond length *L*_e_ is determined by Equation (9), *β*_L_ is a coefficient reflecting the ratio between embedment length to effective bond length and is obtained by Equation (13).

(10) $$V_{u}=0.85\left(1-\frac{\pi R^{2}}{b_{p}L}\right)\varphi_{f}^{0.25}f\prime_{c}^{0.33}\sqrt{L_{per}E_{p}A_{p}}\;(L\geq L_{e},V_{u,\max}<f_{rup}A_{p})$$

(11) $$V_{u}=0.28\beta_{L}\left(1-\frac{\pi R^{2}}{b_{p}L}\right)\varphi_{f}^{0.25}f\prime_{c}^{0.33}\sqrt{L_{per}E_{p}A_{p}}+V_{GPC}\;L<L_{e}$$

(12) $$V_{GPC}=\frac{4.34\tau_{u}\left(e-R\right)t}{1+0.15\left[\frac{b_{p}}{2R}-1.5\frac{\left(b_{p}/2R-1\right)}{\left(b_{p}/2R+1\right)}\theta \right]}$$

(13) $$\beta_{L}=\sin\left(\frac{\pi L}{2L_{e}}\right)$$

Parametric analysis results of SCGPC have been used to validate the suggested empirical equation. The prediction by Equations (10) and (11) are summarized in Table 6, which shows a mean deviation −0.07 between the prediction by the suggested equations and FE simulation. The prediction of empirical equation matches the strength from FE analysis. The precondition of Equations (10) and (11) is GFRP plate thickness *t* < *t*_cr_. When *t* ≥ *t*_cr_, it may relate to the resistance of the concrete dowel, which is not the objective of this paper.

## 6. Conclusions

In this research, sand-coated GFRP plates and GPC pull-out tests were performed. Numerical models of SCGPC were built to evaluate the effect of parameters such as embedment length and radius. Analytic and numerical analyses were made to reveal the shear failure mechanism of SCGPC. The results of this research can be summarized as follows:

1. The shear capacity of SCGPC is considerably larger than that of GPC. The stiffness of SCGPC is determined by the adhesion. The ductility of SCGPC is improved especially when the embedment length meets the effective bond length requirement, which results in the load-slip presenting a yield plateau similar as the steel material.
2. SCGPC has the same characteristics as the sand-coated GFRP plate or rebar. Among the parameters affecting adhesion capacity, it is found that embedment length is the most dominant factor. When the embedment length is larger than effective bond length, the adhesion strength governs the strength of SCGPC; when the embedment length is less than effective bond length, the strength of SCGPC is determined by both the adhesion and GPC shear strength. In the meantime, SCGPC also has the nature of GPC; the shear failure mechanism of SCGPC has a close relation with the radius and the plate’s thickness same as GPC.
3. An empirical equation is suggested to predict the shear strength of SCGPC. The equation solves the strength of SCGPC in two ranges according to the embedment length. The parametric analysis result agrees well with the suggested equation.
4. SCGPC provides an effective alternative connection to GFRP-concrete composite structures. Compared to purely sand-coated GFRP plate, SCGPC has larger ductility. Compared to GPC, SCGPC’s shear strength is considerably improved by sand-coated surface treatment.

These conclusions are drawn under the circumstance of *t* < *t*_cr_. For SCGPC of *t* ≥ *t*_cr_, the failure mechanism needs to be further studied.

## Figures and Tables

**Table 1:** Material property of pultruded GFRP.

Property	Value	Unit	Standard Deviation
Longitudinal tensile strength	430.0	MPa	31.3
Longitudinal tensile modulus	45.5	GPa	4.5
Longitudinal compressive strength	491.4	MPa	54.7
Transverse tensile strength	67.6	MPa	2.8
Transverse tensile modulus	21.7	GPa	1.9
Transverse compressive strength	166.7	MPa	16.9
shear strength	58.4	MPa	10.1
shear modulus	9.8	GPa	0.9

**Table 2:** Pull-out test specimens (unit: mm).

Specimens NO.	Surface Treatment	*R*	*b*	*b*_p_	*L*	Diameter of Penetrating Rebars
SC-P	Sand-coated	-	300	130	300	-
PL15D10	Lubricated	15			300	10
PL20D16	Lubricated	20			300	16
PL25D16	Lubricated	25			300	16

**Table 3:** Ultimate strength of laminas (unit: MPa).

Item	Value
*X*_T_	1335.2
*Y*_T_	955.4
*X*_C_	43.8
*Y*_C_	155.8
*S*_L_	76.0
*S*_T_	76.0

**Table 4:** Predicted ply thickness.

Ply	Angle (°)	Thickness (mm)
1	0	1.7
2	90	0.28
3	±45	0.8
4	0	1.7
5	±45	0.8
6	90	0.28
7	±45	0.44

**Table 5:** Dimensions of parametric specimens (unit: mm).

Specimens NO.	Surface Treatment	R	Embedding Length	Multi-Hole
NS-R25-E300	None	25	300	
NS-R30-E300	None	30	300	
NS-R35-E300	None	35	300	
SC-R25-E300	Sand-coated	25	300	
SC-R30-E300	Sand-coated	30	300	
SC-R35-E300	Sand-coated	35	300	
NS-R25-E200	None	25	200	
NS-R30-E200	None	30	200	
NS-R35-E200	None	35	200	
SP-E200	Sand-coated	-	200	
SC-R25-E200	Sand-coated	25	200	
SC-R30-E200	Sand-coated	30	200	
SC-R35-E200	Sand-coated	35	200	
NS-R25-E150	None	25	150	
NS-R30-E150	None	30	150	
NS-R35-E150	None	35	150	
SP-E150	Sand-coated	-	150	
SC-R25-E150	Sand-coated	25	150	
SC-R30-E150	Sand-coated	30	150	
SC-R35-E150	Sand-coated	35	150	
SC-R25M-E150	Sand-coated	25	150	Two holes
SC-R30M-E150	Sand-coated	30	150	Tow holes

**Table 6:** Validation of the empirical equation.

Specimens NO.	R(mm)	Embedment Length, L(mm)	Strength by FE (kN)	Equations (10) and (11) (kN)	Deviation
SC-R25-E300	25	300	289.6	272.7	−0.058
SC-R30-E300	30	300	262.4	266.3	0.015
SC-R35-E300	35	300	261.5	258.8	−0.010
SC-R25-E200	25	200	235.1	193.7	−0.176
SC-R30-E200	30	200	229.9	191.6	−0.166
SC-R35-E200	35	200	220.9	186.4	−0.156
SC-R25-E150	25	150	182.4	174.2	−0.045
SC-R30-E150	30	150	174.2	171.9	−0.013
SC-R35-E150	35	150	169.9	167.3	−0.016
